# Oxidative Stress Markers Associated with Gingival Inflammatory Status in Children with Leukemia

**DOI:** 10.3390/diagnostics15222915

**Published:** 2025-11-18

**Authors:** Alina Adumitroaie, Larisa Ghemiș, Maria-Alexandra Mârțu, Liliana Georgeta Foia, Catalina Iulia Saveanu, Delia Lidia Salaru, Alina Andronovici, Carmen Delianu, Vasilica Toma

**Affiliations:** 1 Grigore T. Popa University of Medicine and Pharmacy Iasi, Universitatii Street 16, 700115 Iasi, Romania; alina.adumitroaie@umfiasi.ro (A.A.); larisa.danaila@umfiasi.ro (L.G.); maria-alexandra.martu@umfiasi.ro (M.-A.M.); catalina.saveanu@umfiasi.ro (C.I.S.); deliasalaru@gmail.com (D.L.S.); vasilica.toma@umfiasi.ro (V.T.); 2 Division of Medical Analyses Laboratory, Clinical Emergency Hospital St. Spiridon, 700111 Iasi, Romania; carmendelianu@gmail.com; 3 Institute of Cardiovascular Diseases, 50 Carol I Avenue, 700503 Iasi, Romania; 4 PerioClinic Private Practice, 4 Basota Avenue, 700802 Iasi, Romania; alina_andronovici@yahoo.com

**Keywords:** gingival disease, gingival pathology, gingival inflammation, children, leukemia, oxidative stress

## Abstract

**Background/Objectives**: This study aimed to investigate specific biomarkers of oxidative stress within gingival crevicular fluid (GCF) and plasma obtained from children with leukemia compared to healthy subjects, in relation to the oral hygiene status and gingival inflammatory status, in order to identify a possible association linking childhood leukemia with gingival inflammation. **Methods**: The study comprised biomarker analysis from 97 children divided into two groups: 47 leukemia subjects and 50 systemically healthy children in the control group. The GCF and plasma specimens were analyzed to determine values of 8-OHdG (8-hydroxydeoxyguanosine) and SOD (superoxide dismutase) using ELISA (enzyme-linked immunosorbent assay) techniques, while MDA (malondialdehyde) values were measured through colorimetry. **Results**: We found elevated plasma expressions of all investigated biological parameters among leukemic children relative to the control group. GCF measurements highlighted raised 8-OHdG and SOD in leukemic individuals, while MDA recorded no significant shift between the groups. The statistical analysis also revealed a possible GCF and plasma SOD levels associated with the oral hygiene and gingival inflammatory status. **Conclusions**: The increased expression of oxidative stress markers we found in children with leukemia underlines the heightened inflammatory and oxidative burden in this category of population, yet additional studies are needed to clarify the intricate relation between systemic oxidative stress, oral biomarkers and gingival health outcomes in children, especially in children with critical systemic alterations such as leukemia.

## 1. Introduction

Oxidative stress appears whenever protective antioxidant systems can no longer neutralize the actions of free radicals, phenomena which has been proven to act in a direct or indirect manner including in the oral cavity [1,2,3].

Inflammatory disease progression significantly correlates with the activity of oxygen reactive species, and the scientific literature is becoming increasingly focused on determining the implications of oxidative stress, especially in the early diagnosis and prognostic challenges, as well as in the evolution of certain pathologies [4]. Although several issues remain unclear in the etiopathogenesis and management of periodontal diseases, most studies endorse oxidative stress as a significant factor influencing both [5].

The importance of oxidative stress as a determinant etiopathogenic factor for periodontal disease is linked to the pro- and anti- inflammatory balance found in the periodontal tissues. Most researchers have demonstrated the direct involvement of oxidative stress in the gradual decay of extracellular matrix components of the periodontal attachment tissues [6,7].

Various systemic conditions and diseases can influence the inflammatory status of gingival and periodontal tissues, including but not only: obesity, auto-immune diseases, hormonal imbalance, chronic kidney disease or immunosuppressive therapy, all potentially altering the overall stomatognathic health and, particularly, periodontal status [8,9,10,11]. Having said this, some literature reviews have described oxidative stress markers as essential pieces in understanding the pathophysiological mechanisms of inflammatory diseases [12].

The most frequent method of oxidative stress evaluation consists of identification of oxidative stress products in lipids, proteins or DNA, of which malondialdehyde (MDA), superoxide dismutase (SOD), glutathione peroxidase (GPx), and 8-Hydroxydeoxyguanosine (8-OHdG) have been extensively explored [13].

Malondialdehyde (MDA) is a marker of oxidative damage resulting from lipid peroxidation, a reaction triggered by free radicals, which leads to tissue impairment [14]. MDA concentration seems to be directly related to oxidative stress [15,16]. 8-Hydroxydeoxyguanosine (8-OHdG) is a predominant form of free radical-induced oxidative lesions of genetic material; some authors underline its role as a pivotal marker for measuring the effect of endogenous oxidative damage to DNA [17]. Superoxide dismutase (SOD) seems to reflect the body’s defensive response to oxidative stress, as SOD activity initially increases as an adaptive up-regulation to elevated free radicals, with subsequent decrease after sustained or excessive oxidative stress, reflecting enzyme consumption, inactivation or exhaustion of the antioxidant arsenal [18,19].

The majority of studies have been conducted on adult patients, while few are focused on the correlation of salivary oxidative stress biomarkers and dental or periodontal status in children and adolescents [20,21,22,23,24,25,26,27,28]. Moreover, the results of these few studies on children and adolescents are often contradictory, which can be attributed either to different saliva or GCF collection techniques, various storage methods or marker identification, as well as different approaches in periodontal disease classification [29]. To complicate the issue even more, if there is a general condition or disease the treatment can lead to an alteration of the salivary composition, as well as modifications of oxidative stress biomarkers [30,31,32,33].

Currently, the literature has undergone a shift from the periodontal severity evaluation based solely on clinical measurements, to identifying new diagnostic tools, which can possibly offer a prognostic over the tissue destruction extension and also the inflammation degree. Therefore, oxidative stress parameters found in serum, saliva or gingival crevicular fluid might be key factors in the early diagnosis of periodontal alteration [34,35,36,37].

Skutnik-Radziszewska and Zalewska have concluded, in their 2020 study, that oxidative stress markers have a potential role in the screening, diagnosis and monitoring of inflammatory disease progression, but their routine clinical use could not be inserted in clinical practice yet [38]. The current literature is scarce regarding the evaluation of oxidative stress markers in children with leukemia and gingival inflammatory status, and only a few studies have addressed this particular association.

The aim of this study was to evaluate oxidative stress markers malondialdehyde, superoxide dismutase and 8-Hydroxydeoxyguanosine values in plasma and gingival crevicular fluid in children with leukemia and assess whether there is a correlation with the oral hygiene and gingival inflammatory status.

## 2. Materials and Methods

The present study was conducted in accordance with the Declaration of Helsinki, following approval of the Ethical Committee of the “Grigore T. Popa” University of Medicine and Pharmacy from Iasi (approval no. 477/16 September 2024) and the Ethical Committee of the “St. Mary” Clinical Emergency Hospital for Children in Iasi (approval no. 34161/14 October 2024).

The study initially included 99 children, aged between 3 and 18 years old, monitored within the “St. Mary” Clinical Emergency Hospital for Children in Iasi between October 2024 and May 2025. Out of all children, 49 were included in the study group, with a confirmed diagnosis of leukemia (28 boys and 21 girls); the other 50, comprising children without an oncological diagnosis, were allocated to the control group and were monitored in the hospital for other non-inflammatory conditions (21 boys and 29 girls).

The inclusion criteria consisted of children up to 18 years of age, with specific requirements for each group. For the study group, children with any form of leukemia (acute lymphoblastic leukemia, acute myeloid leukemia or chronic myeloid leukemia) were enrolled at any stage of oncological treatment, regardless of periodontal status, provided they had not been submitted to any specific periodontal treatment in the 6 months prior to examination. For the control group, children without leukemia who had not undergone any specific periodontal treatment in the 6 months prior to the examination were included.

For both groups, exclusion criteria resided on subjects over 18 years of age or who had undergone specific periodontal treatment in the 6 months prior to the examination.

**Figure 1:**
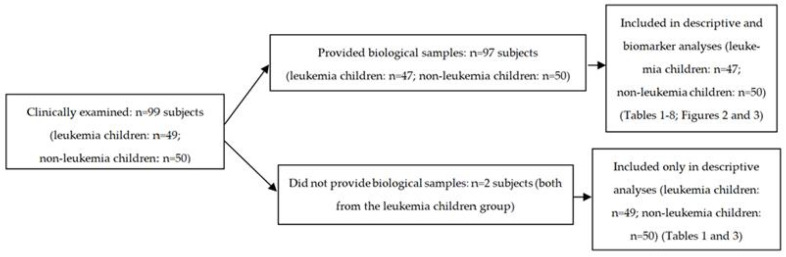
Flow diagram of participants in the study.

Clinical examination of all 99 subjects (study group: *n* = 49; control group: *n* = 50) was conducted, but biological samples were collected from only 97 children, as two subjects from the study group declined sample collection. Therefore, the biomarker analysis set comprised 97 subjects (*n* = 47 leukemia children; *n* = 50 subjects without leukemia). The two children with leukemia who had declined biological sampling were nonetheless included in the descriptive analysis of clinical, oral hygiene status and gingival inflammation status data, and were only excluded from the biomarker measurements and related statistical tests. No data imputation was applied (Figure 1).

The clinical examination of all young patients was performed by a periodontal specialist who had been previously calibrated for the assessment of oral hygiene and gingival inflammation using the Simplified Oral Hygiene Index (OHI-S) modified after Greene and Vermillion and the Gingival Index (GI) according to Löe and Silness. The OHI and GI scoring systems and category cutoffs are presented in Table 1. Although quantitative reproducibility statistics were not computed in this study, the examiner underwent calibration training under the supervision of an experienced periodontist, with repeated assessments until consistent scoring was achieved.

The subsequent steps (sample collection and oxidative stress markers analysis) are presented in Table 2.

## 3. Results

### 3.1. Descriptive Data

The descriptive demographical and clinical characteristics of subjects in both groups are presented in Table 3. The children included in this study were between 3 and 18 years old, with mean values of 12.16 (±0.51) years in the control group and 8.71 (±0.53) years in the study group. The gender distribution of subjects was balanced, with 49 boys (49.5%) and 50 girls (50.5%). When the groups were analyzed comparatively, a higher percentage of boys was seen in the study group compared to the control group, where girls were more prevalent.

Regarding the environment distribution of children, rural areas were more represented in both groups, with 76% of subjects in the control group and 61.2% in the study group belonging to a rural environment.

We evaluated the distribution of children in both groups according to their oral hygiene status. Out of the total number of subjects, children with poor oral hygiene were only seen in the study group (7 out of 7 cases), unsatisfactory oral hygiene was found mostly in the study group (17 out of 26 cases), while satisfactory and good oral hygiene status was mainly recorded in the control group.

The evaluation of the gingival inflammatory status by group pointed out severe gingival inflammation exclusively in subjects from the study group (13 out of 13 children), whereas children with moderate gingival inflammation were mostly found within the study group (14 out of 23 cases) and subjects with mild gingival inflammation were mainly found in the control group (41 out of 63 children).

When analyzing the study group, we observed that most of the children were affected by acute lymphoblastic leukemia (ALL—approximately 75.5% of cases), with a predominance of the maintenance phase of treatment in 55.1% of all subjects being noted.

### 3.2. Oxidative Stress Biomarkers Analysis

Table 4 summarizes the mean local (GCF) and systemic (plasma) biological parameters’ levels measured in the study and control groups as well as the comparative description between these values.

The results indicate that all three biomarkers evaluated in plasma presented statistically significant differences between the two groups, with constant augmented values in the study group compared to the control group. In gingival crevicular fluid, 8-OHdG and SOD had a significant distinction between the study and the control group, while MDA, one of the most explored lipid peroxidation products in periodontitis, did not present statistically significant differences between the groups.

**Figure 2:**
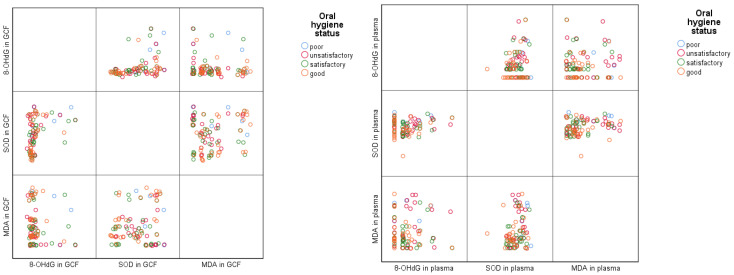
Graphic distribution of the association between oral hygiene status and oxidative stress markers in GCF and plasma.

We used matrix scatterplots to analyze the association between oral hygiene status and the oxidative stress markers evaluated in GCF and plasma, and the results are depicted in Figure 2. In the case of GCF samples, we observed slightly elevated levels of 8-OHdG in subjects with poor or unsatisfactory oral hygiene status; for MDA and SOD levels, the distributions are more uniform, but children with good oral hygiene seem to have medium levels of the analyzed markers, while those with unsatisfactory oral hygiene status appear in the extreme levels of the evaluated markers. Regarding plasma samples, values of 8-OHdG and MDA seem to be more increased and have a greater variability in poor and unsatisfactory oral hygiene groups. Plasma values of SOD seem more uniformly distributed and do not distinguish clear differences between oral hygiene groups.

**Figure 3:**
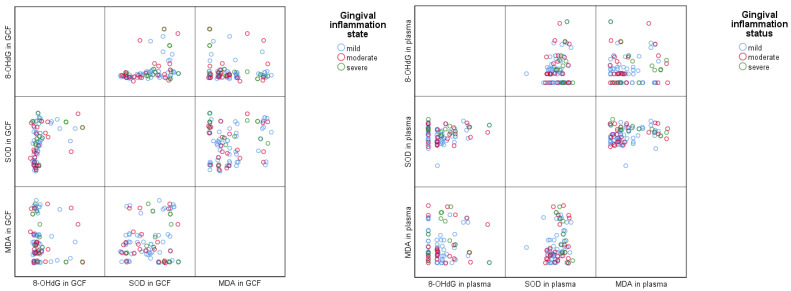
Graphic distribution of the association between gingival inflammation status and oxidative stress markers in GCF and plasma.

To identify possible associations between gingival inflammation status and the local and systemic levels of the evaluated oxidative stress markers (8-OHdG, MDA and SOD), scatterplot matrices were used (Figure 3). For gingival crevicular fluid samples, the scatterplot highlighted a greater dispersion in the severe inflammation group for 8-OHdG and MDA values, while the SOD values were similarly dispersed between the three groups, suggesting that there were no large variations for this biomarker. For the plasma biomarkers, the scatterplot shows relatively similar values between groups, with the exception of 8-OHdG and MDA, which appear higher in moderate and severe inflammation groups, compared with the mild inflammation group (Figure 3). Overall, the graphic suggests that gingival inflammation severity is associated with an increase in 8-OHdG and MDA values, while SOD remains relatively constant.

The distribution of children in the study and control groups ensured an adequate variability across oral hygiene status and gingival inflammation levels, allowing the general linear model (GLM) to assess their independent and combined effects on the oxidative stress biomarkers (8-OHdG, SOD, MDA). However, the small number of participants with poor hygiene (*n* = 7) or severe inflammation (*n* = 13) should be acknowledged as a limitation when interpreting interaction effects (OHI × GI or higher-order terms) (Table 5).

A multivariate analysis of variance (MANOVA) was conducted to examine the combined effects of clinical and demographic factors on oxidative stress biomarkers (8-OHdG, SOD and MDA) (Table 6). The results show a significant multivariate main effect of the group (Pillai’s Trace = 0.390, F (6, 68) = 7.25, sig 0.001, η^2^*p* = 0.39), indicating that the overall oxidative stress markers differed significantly between children with leukemia and healthy controls. This effect was large in magnitude, with high statistical power (0.999), confirming robust group differences across the biomarker set. In contrast, no significant multivariate effects were observed for gender, oral hygiene status (OHI), gingival inflammation (GI) or age (all *p* > 0.05). Furthermore, interaction effects such as Gender × OHI, Gender × GI, Group × OHI and Group × GI were not statistically significant, suggesting that the effect of leukemia status on oxidative stress does not depend on gender or oral health categories.

The results of the general linear model (GLM) framework that reach the established significance level (α = 0.10) are presented in Table 7 and suggest biologically relevant main effects and interactions, highlighting systematic differences between leukemia children and the control group, while the other factors did not show significant influences on oxidative stress markers (see Appendix A, Table A1).

The GLM analysis revealed significant between-group differences for several oxidative stress biomarkers. For 8-OHdG, both in gingival crevicular fluid (GCF) (F = 9.38, *p* = 0.003, η^2^*p* = 0.114) and in plasma (F = 4.21, *p* = 0.044, η^2^*p* = 0.055), values were significantly higher in leukemia children compared to controls. These effects indicate that approximately 11% (GCF) and 6% (plasma) of the variability in 8-OHdG levels are explained by disease status. Similarly, SOD activity showed highly significant differences between groups, both in GCF (F = 15.66, *p* < 0.001, η^2^*p* = 0.177) and in plasma (F = 20.94, *p* < 0.001, η^2^*p* = 0.223). These large effect sizes suggest that group explains between 18% and 22% of the variance, confirming a consistent oxidative imbalance in leukemia children. For MDA, age was a significant predictor for GCF values (F = 4.97, *p* = 0.029, η^2^*p* = 0.064), indicating a slight but measurable age-related increase. In plasma, MDA levels were marginally higher in leukemia children compared to controls (F = 3.33, *p* = 0.072, η^2^*p* = 0.044), suggesting a near-significant trend at the 10% level.

The GLM analysis also showed that three interaction effects reached marginal significance at the 10% threshold, suggesting possible moderating influences between local oral parameters and demographic factors (Table 8). For GCF values of 8-OHdG, the interaction between gender and gingival inflammation (GI) was significant for 10% (F = 2.49, *p* = 0.090, η^2^*p* = 0.064). This indicates that the association between gingival inflammation and oxidative damage may differ between boys and girls, as inflammation may reflect gender-specific susceptibility to local inflammatory stress. For plasma values of 8-OHdG, the OHI × GI interaction (F = 2.07, *p* = 0.067, η^2^*p* = 0.146) suggests that oral hygiene and gingival inflammation may jointly influence systemic oxidative stress. Finally, the Gender × OHI interaction for SOD values in plasma (F = 2.60, *p* = 0.058, η^2^*p* = 0.097) indicates that the relationship between oral hygiene and SOD activity might differ by gender.

The interactions identified at α = 0.10 were considered exploratory due to the limited sample size and the unbalanced distribution across oral health categories. Therefore, no post hoc or simple effects analyses were performed, and these interactions were only discussed descriptively to avoid overinterpretation.

Since treatment phase and diagnosis subtype are potential confounders that could affect both treatment-related symptomatology and outcome of the oxidative markers measurements, we conducted a sensitivity analysis including only leukemia patients in the maintenance treatment phase (*n* = 27), related to the same control group, to assess whether treatment phase confounded the main results (Table 9). The GLM (adjusted for age, gender, oral hygiene and gingival inflammation) showed no significant effects of oral health parameters or gender on any of the evaluated biomarkers (*p* > 0.05). Age was slightly associated only with plasma 8-OHdG (F = 4.54, *p* = 0.046), while all other associations, including those for SOD and MDA, were non-significant. This confirms that the previously observed differences in oxidative stress markers between leukemia and control groups were not driven by treatment-phase heterogeneity. The sensitivity analysis therefore supports the robustness of the main findings.

## 4. Discussion

The literature data highlights an ascendant trend of leukemia within the pediatric population, especially in younger age groups [39,40,41,42,43]. In our study, subjects affected by different forms of leukemia were mostly found in the 5–10 years old age group, with a mean age of 8.71 (±0.53), consistent with the recent findings of other authors [44].

Studies report that boys are more likely to be affected by leukemia, as compared to girls [45,46]. The gender distribution of subjects in the leukemia group of our study seems to be similar to the percentages found in the literature, with 57.1% of the affected children being boys and 42.9% girls.

Considering the environmental distribution of children with leukemia, the literature offers inconsistent results. For example, some authors found higher prevalences of the oncologic disease in children from urban areas [47,48,49], while others found that most children with leukemia came from rural areas [50,51]. In our study, 61.22% of children with leukemia resided in rural areas.

The type of leukemia is also a variable that can define geographic areas or various populations, most studies reporting a higher prevalence of acute lymphoblastic leukemia (ALL) among children with this oncologic pathology [52,53,54,55]. The results of our study are consistent with these findings, acute lymphoblastic leukemia being the most frequent subtype encountered in the selected and enrolled group.

The existing literature addressing the oral hygiene status of children with leukemia is rather scarce. However, several studies have evaluated this aspect, either descriptively or experimentally, after the implementation of an oral care protocol. Their findings vary depending on the treatment phase of the leukemia and whether oral hygiene measures were applied or not. However, most papers report poor oral hygiene in children with acute lymphoblastic leukemia, with alterations of the oral health status in different stages of the chemotherapy treatment [30,56,57,58]. The subjects in our study were not submitted to any oral care protocol measures, and the results point out that the oral hygiene status of children with leukemia was mostly poor and unsatisfactory.

Our findings underline the necessity of preventive management and the implementation of oral health care protocols in children with leukemia, as suggested by previous researchers [59]. Moreover, there are certain studies that report an improvement of oral hygiene status in leukemia children following different oral hygiene regimens or different oral care protocols during oncological treatment [60,61,62,63,64,65]. These results emphasize the importance of standardized preventive protocols but also reinforce the need for individualized dental management of children with leukemia.

Among the most commonly reported manifestations of leukemia and its therapy, the literature reports gingival changes [66,67]. The largest studies show that children with leukemia encounter altered periodontal status, with increased gingival inflammation status [56,68,69,70]. In our study, the results show that subjects in the leukemia group had an altered gingival state, with mostly severe and moderate gingival inflammation status, compared to systemically healthy children. These findings highlight the need for implementation of oral health care protocols in this vulnerable segment of the population in order to improve gingival inflammation and the periodontal health status, as suggested by other studies [61,64].

In the context of oral hygiene and gingival inflammation, oxidative stress plays a key role, and the assessment of oxidative stress biomarkers like 8-OHdG, MDA or SOD can offer valuable insight into the underlying biological mechanisms [38]. However, research is limited in respect to monitoring these biomarkers in children in general, and, in particular, in children with leukemia.

One study conducted on young patients has reported that some specific oxidative markers have correlated with oral hygiene, dental and periodontal status. The authors have underscored the possibility of variabilities between biomarkers’ manifestation in children and adult, linked to differences in oral hygiene routines, hormonal imbalances or other physiological conditions [24].

8-OHdG has been described by some researchers as an oxidative stress marker that can be used in the early diagnosis and also as a monitoring tool in the treatment of severe periodontal disease [71,72,73]. Other authors have observed that 8-OHdG values increase even before the first clinical signs of periodontal disease [74].

Moreover, several studies have highlighted the presence of increased values of 8-Hydroxydeoxyguanosine (8-OHdG) in the GCF or saliva of individuals with periodontal impairment, significantly correlating it with clinical parameters of periodontal inflammation [35,71,74,75,76,77,78,79,80]. Some authors observed a correlation between elevated serum and salivary levels of 8-OHdG and the presence of periodontal disease in individuals with diabetes [81]. Nevertheless, searching the literature database, we have not found studies evaluating this marker in children and adolescents, either systemically healthy or with an underlying medical condition.

In our study, we observed increased values of 8-OHdG, both in plasma and GCF, in children with leukemia as compared to subjects in the control group. The statistical analysis revealed that poor oral hygiene was likely associated with higher plasma 8-OHdG only in children with moderate and severe gingival inflammation, implying that local inflammatory status can amplify systemic oxidative imbalance.

Studies associate high levels of MDA with oxidative stress in periodontal disease [82,83,84,85,86,87,88]. Some authors have reported significantly more increased values of MDA in the crevicular fluid of periodontal disease patients, compared to healthy individuals [89,90,91].

It seems that the gingival crevicular fluid MDA value can be a useful tool in differentiating between different severities of periodontal disease and in identifying a normal periodontal status [92]. One study indicates a lack of statistically significant differences between MDA levels in individuals with periodontal disease and healthy subjects, but correlates high levels of MDA with the sharpness of alterations in periodontal disease; moreover, the authors identified a connection between high levels of MDA in GCF and the activity of free radicals in periodontal inflammation [93].

Most research centered on adults have proportionally correlated the presence of MDA in gingival fluid and blood samples with different degrees of periodontal disease severity [94,95]. In the pediatric population, increased MDA levels were observed in children with hepatitis C, sleep disorders or general growth impairments [96,97,98]. One relatively recent work correlates high values of MDA with the progression of periodontal disease, authors reporting increased levels of this biomarker in children with moderate and severe gingivitis compared to those with mild gingivitis [28].

The results of our study display slightly increased levels of plasma MDA in children with leukemia compared to systemically healthy children. In gingival crevicular fluid, however, MDA values were not significantly different between the two study groups. Regarding the association between this biomarker and oral health or gingival inflammation status, the statistical analysis also showed no relevant significance; however, MDA values in GCF rather seem to increase according to the subject’s age. These findings may be attributed to physiological or behavioral differences in oral hygiene habits or to varying oxidative stress responses between patients of different ages; however, further investigations are needed to better understand the underlying mechanisms.

Considering the correlation between the presence of SOD in gingival crevicular fluid and periodontal disease, there are some studies performed on adult subjects that confirm an increased activity of superoxide dismutase (SOD) in periodontal disease patients compared to healthy individuals. This increased activity correlates with the clinical severity of the inflammation, evaluated by clinical attachment levels, bleeding on probing, probing depth, gingival index or bacterial plaque index [38,90,99].

Akalin et al. [100] have associated high values of SOD with the presence of periodontitis, suggesting that SOD activity increases with the progression of periodontal inflammation. Other authors have also reported increased levels of SOD activity in individuals with periodontal disease compared to healthy people, correlating the severity of the periodontal disease with a gradual decrease in antioxidant concentration [99,101]. Thus, SOD most likely acts as an antioxidant defense mechanism that exacerbates during periodontal disease. Some studies have reported significant improvements of this salivary oxidative stress marker after applying certain periodontal treatment methods [102,103].

There are only few available studies on pediatric population, but most papers analyze the presence of SOD in different systemic disease. Some authors reported increased values of SOD in children with juvenile idiopathic arthritis [104], while decreased levels were observed in children with autism, acute asthma episodes or those with general growth deficiencies [105,106,107]. Obradovic (2020) has evaluated oxidative stress parameters in adolescents with gingival inflammation, reporting minimal changes in SOD values, both in periodontal disease individuals and in healthy subjects [26]. The author underlined that some oxidative stress biomarkers could represent useful instruments in the control of periodontal disease evolution, but further research is necessary in order to understand the complex effects of oxygen reactive species in the tissue degradation process [26].

The results of our study are similar to other findings in the literature, showing increased values of SOD, both in plasma and in the gingival crevicular fluid of children with leukemia, as compared to subjects in the control group. Moreover, GCF and plasma levels of SOD tend to be increased in leukemic children with poor oral hygiene and severe gingival inflammation.

Almost all the oxidative stress markers we evaluated differed significantly between the study and control groups, with the exception of MDA values in gingival crevicular fluid. The multivariate analysis of variance (MANOVA) showed that the increased biomarker values we observed in children with leukemia may suggest that the systemic disease exerts a stronger influence on oxidative stress than local oral health parameters or demographic factors. The findings indicate that increased oxidative imbalance in children with leukemia is primarily attributable to the disease and its systemic effects, rather than to differences in oral hygiene or gingival inflammation.

Overall, these results suggest that belonging to the leukemia group seems to be the dominant determinant of oxidative stress biomarker values, particularly for 8-OHdG and SOD, while age seems to exert a minor independent effect on MDA. When the analysis was restricted to subjects in the maintenance phase of the leukemia treatment, no significant group effects were recorded for any biomarker, suggesting that the large oxidative stress level observed in the full sample were not only attributable to differences in treatment phases.

There are several limitations to be considered when interpreting this study’s results. First, the relatively small sample size may affect the significance of the results; second, there were no additional statistical adjustments made to isolate the independent effect of oral hygiene status on the oxidative stress markers between groups. Oral hygiene, as well as subsequent gingival inflammation, could be influenced by different variables between groups, related to the systemic condition of children in the leukemia group or various oral hygiene practices in the two groups. In this study, we reported our observed findings; however, in order to minimize bias and to more accurately clarify the genuine relationship between leukemia and the oxidative stress response in the oral environment, adjusting for oral hygiene status would provide valuable insight. When comparing our results with the existing literature, it is noteworthy that most studies evaluate oxidative stress markers in gingival fluid and plasma of subjects with varying degrees of periodontal disease, whereas only one identified study [28] assessed these biomarkers in relation to gingivitis—similar to our study’s focus on oral hygiene and gingival inflammation status. Another limitation of this study is that oral mucositis was not formally evaluated for the children in the study group; although it was noted in three patients belonging to the severe gingival inflammation category, it was not considered in the evaluation.

Although the observed increase in oxidative stress markers in our study underlines the heightened inflammatory and oxidative burden in this vulnerable population, additional studies are needed to clarify the intricate relation between systemic oxidative stress, oral biomarkers and gingival health outcomes in children with leukemia.

Given the complex mechanism of oxidative stress and its underlying implications in the progression of gingival inflammation, further research is needed to clarify this relationship in pediatric populations, and particularly among children with leukemia and gingival-periodontal impairment.

## 5. Conclusions

8-OHdG, SOD and MDA recorded elevated plasma levels in children with leukemia compared to systemically healthy controls. In gingival crevicular fluid, both 8-OHdG and SOD levels were significantly increased in the leukemia group, whereas MDA values did not differ significantly between the two groups.

These results offer a base for further explorations in future longitudinal studies and indicate that 8-OHdG, SOD and MDA could serve as potential adjunctive oxidative stress biomarkers of oral inflammatory burden in medically compromised pediatric patients.

## Figures and Tables

**Table 1:** Criteria, calculation methods and interpretation of clinical indices used.

Index	Examined Teeth	Component	Criteria	Calculation Method	Interpretation
Simplified Oral Hygiene Index (OHI-S) modified after Greene and Vermillion (1964)	1.6 (buccal) 1.1 (labial) 2.6 (buccal) 3.6 (lingual) 3.1 (labial) 4.6 (lingual) Or their primary substitutes: 5.5 (buccal) 5.1 (labial) 6.5 (buccal) 7.5 (lingual) 7.1 (labial) 8.5 (lingual)	DI-S (Debris Index-Simplified)	0—No debris or stain	OHI-S = DI-S + CI-S Where DI-S = mean of DI-S scores/number of teeth; CI-S = mean of CI-S scores/number of teeth	0–1: Good oral hygiene status 1.1–2: Satisfactory oral hygiene status 2.1–3: Unsatisfactory oral hygiene status 3.1–6: Poor oral hygiene status
			1—Soft debris on ≤1/3 of tooth surface		
			2—Soft debris on >1/3 but ≤2/3 of tooth surface		
			3—Soft debris on >2/3 of tooth surface		
		CI-S (Calculus Index-Simplified)	0–No calculus		
			1—Supragingival calculus on ≤1/3 of tooth surface		
			2—Supragingival calculus on >1/3 but ≤2/3 of tooth surface or flecks of subgingival calculus		
			3—Heavy supragingival calculus on >2/3 of tooth surface or continuous subgingival band		
Gingival Index (GI) according to Löe and Silness (1963)	Mesial, distal, buccal/labial and lingual surfaces of 1.6, 1.1, 2.6, 3.6, 3.1, 4.6 or their primary substitutes (5.5, 5.1, 6.5, 7.5, 7.1, 8.5)	GI	0—Normal gingiva, no inflammation	GI score per tooth = Mean of GI scores for all 4 surfaces/4 GI score per individual = Mean of GI scores for all teeth/number of teeth	0.1–1: Mild gingival inflammation 1.1–2: Moderate gingival inflammation 2.1–3: Severe gingival inflammation
			1—Slight color change, slight edema, no bleeding on probing (mild inflammation)		
			2—Redness, edema, bleeding on probing (moderate inflammation)		
			3—Marked redness, hypertrophy, ulceration, tendency to spontaneous bleeding (severe inflammation)		

**Table 2:** Protocol of MDA, SOD and 8-OHdG evaluation in gingival crevicular fluid and plasma.

Evaluation Protocol of MDA, SOD and 8-OHdG		
Fluid	GCF	Plasma
Sample collection	Absorbent paper cones; Eppendorf tubes containing 200 µL PBS (phosphate-buffered saline solution)	EDTA-containing tubes
Algorithm	Isolation of the selected teeth (maxillary or mandibular incisors, maxillary or mandibular first permanent molars) using cotton rolls Gentle removal of any visible supragingival plaque using cotton rolls Insertion of the sterile absorbent paper cone for 30 s into the gingival sulcus at the mesial sites of the selected teeth Insertion of the paper cones in sterile Eppendorf tubes and transportation on ice up to the Periotron calibrated device (Periotron 8000, Oraflow Inc., New York, NY, USA), for the GCF volume measurement with subsequent storage at −80 °C until biological markers analysis	Routine blood sampling during child’s in-hospital monitoring; immediate centrifugation at 1000× *g* 4 °C for 15 min; storage of the obtained plasma aliquots in Eppendorf tubes at −80 °C until parameters evaluation
Measurements	Sample thawing at analysis time; preparation of all reactive agents and standards according to the manufacturers’ instructions; analysis of SOD and 8-OHdG using ELISA (enzyme-linked immunosorbent assay) kits (Elabscience^®^, Bionovation Inc., Houston, TX, USA); analysis of MDA using a colorimetric kit (Elabscience^®^, Bionovation Inc., Houston, TX, USA) and a semi-automatic analysis device (BioSystems BTS-350, BioSystems S.A., Barcelona, Spain)	
Statistical analysis	All statistical analyses were performed using IBM SPSS Statistics, version 29.0 (IBM Corp., Armonk, NY, USA) (see Appendix A).	

**Table 3:** Demographical and clinical characteristics of individuals in the study and control groups.

Characteristic *	Study Group (*n* = 49)	Control Group (*n* = 50)
Age (years; mean ± SD)	8.71 (±0.53)	12.16 (±0.51)
Gender (*n* [% of group])		
Male	28 [57.1]	21 [42.0]
Female	21 [42.9]	29 (58.0)
Environment (*n* [% of group])		
Urban	19 [38.8]	12 [24.0]
Rural	30 [61.2]	38 [76.0]
Oral hygiene status (*n* [% of group])		
Poor	7 [14.3]	0 [0.0]
Unsatisfactory	17 [34.7]	9 [18]
Satisfactory	14 [28.6]	20 [40]
Good	11 [22.4]	21 [42]
Gingival inflammation status (*n* [% of group])		
Mild	22 [44.9]	41 [82.0]
Moderate	14 [28.6]	9 [18.0]
Severe	13 [26.5]	0 [0.0]

* Demographical characteristics and clinical examination included all subjects (*n* = 99; leukemia children: *n* = 49; children without leukemia: *n* = 50).

**Table 4:** Comparative description of oxidative stress biomarkers values in GCF and plasma of children in the study and control groups.

	Oxidative Stress Biomarker (Unit) *	Group	*n*	Mean	Std. Deviation	Std. Error Mean
Plasma values	8-OHdG (ng/mL)	Control	50	20.659	18.967	2.682
		Study	47	43.840	42.739	6.234
	SOD (U/mL)	Control	50	6.095	0.234	0.033
		Study	47	6.359	0.163	0.023
	MDA (nmol/mL)	Control	50	1.723	1.380	0.196
		Study	47	3.098	2.443	0.356
GCF values	8-OHdG (ng/mL)	Control	50	1.228	0.336	0.047
		Study	47	2.714	2.316	0.337
	SOD (U/mL)	Control	50	6.091	0.221	0.031
		Study	47	6.296	0.172	0.025
	MDA (nmol/mL)	Control	50	2.427	2.108	0.298
		Study	47	2.460	2.210	0.322

* GCF = gingival crevicular fluid; 8-OhdG = 8-Hydroxydeoxyguanosine; SOD = superoxide dismutase; MDA = malondialdehyde. These biomarkers were evaluated on subjects who provided biological samples (*n* = 97; leukemia children: *n* = 47; children without leukemia: *n* = 50).

**Table 5:** Variables used in the statistical analysis.

Variable	Value	Label (Category)	*n*
Gender	1	Male	47
	2	Female	50
Group	0	Control	50
	1	Study	47
Oral hygiene status (OHI category)	0	Poor	7
	1	Unsatisfactory	26
	2	Satisfactory	32
	3	Good	32
Gingival inflammation status (GI category)	1	Mild	61
	2	Moderate	23
	3	Severe	13

**Table 6:** Results of the MANOVA test.

Effect	Multivariate Test	Value	F	Hypothesis df	Error df	Sig. (*p*)	Partial η^2^	Observed Power
Intercept	Pillai’s Trace	0.997	4338.12	6	68	0.001	0.997	1.000
Gender	Pillai’s Trace	0.050	0.59	6	68	0.735	0.050	0.22
Group (Study and Control)	Pillai’s Trace	0.390	7.25	6	68	0.001	0.390	0.999
Oral hygiene status (OHI)	Pillai’s Trace	0.270	1.16	18	210	0.302	0.090	0.78
Gingival inflammation (GI)	Pillai’s Trace	0.099	0.60	12	138	0.840	0.050	0.33
Age	Pillai’s Trace	0.102	1.29	6	68	0.275	0.102	0.47
Gender × OHI	Pillai’s Trace	0.302	1.31	18	210	0.186	0.101	0.84
Gender × GI	Pillai’s Trace	0.197	1.26	12	138	0.251	0.099	0.69
OHI × GI	Pillai’s Trace	0.434	0.95	36	438	0.556	0.072	0.90
Status × OHI	Pillai’s Trace	0.157	0.98	12	138	0.468	0.079	0.55
Status × GI	Pillai’s Trace	0.056	0.68	6	68	0.669	0.056	0.25

**Table 7:** GLM results showing significant main effects (*p* < 0.10).

Dependent Variable	Source	F	*p*-Value	Partial η^2^	Adjusted R^2^ (Model)	Interpretation
8-OHdG (GCF)	Group	9.375	0.003	0.114	0.190	Higher levels in study group
8-OHdG (Plasma)	Group	4.209	0.044	0.055	0.090	Higher levels in study group
SOD (GCF)	Group	15.661	<0.001	0.177	0.213	Higher levels in study group
SOD (Plasma)	Group	20.938	<0.001	0.223	0.244	Higher levels in study group
MDA (GCF)	Age (years)	4.973	0.029	0.064	−0.019	Slight age-related increase
MDA (Plasma)	Group	3.330	0.072	0.044	0.123	Marginally higher in study group

**Table 8:** Significant interaction effects identified by the GLM (α = 0.10).

Dependent Variable	Interaction	F	*p*-Value	Partial η^2^	Note
8-OHdG (GCF)	Gender × Gingival inflammation (GI)	2.486	0.090	0.064	Suggests gender-specific GI patterns; probe simple effects
8-OHdG (Plasma)	Oral hygiene (OHI) × GI	2.073	0.067	0.146	OHI effect varies by GI level; post hoc contrasts recommended
SOD (Plasma)	Gender × OHI	2.603	0.058	0.097	OHI relates to SOD differently by gender; examine simple slopes

**Table 9:** Synthetic results of the sensitivity analysis restricted to maintenance-phase leukemia subjects.

Dependent Variable	Source	F	*p*-Value	Partial η^2^	Adjusted R^2^ (Model)	Interpretation
8-OHdG (GCF)	Age	0.73	0.404	0.037	−0.125	No significant effect
8-OHdG (Plasma)	Age	4.54	0.046	0.193	0.066	Slight age-related increase
SOD (GCF)	–	—	>0.05	—	−0.308	Non-significant across all factors
SOD (Plasma)	–	—	>0.05	—	−0.233	Non-significant across all factors
MDA (GCF)	–	—	>0.05	—	−0.153	No effect detected
MDA (Plasma)	–	—	>0.05	—	−0.064	No effect detected

## Data Availability

The original contributions presented in this study are included in the article. Further inquiries can be directed to the corresponding author.

## Abbreviations

The following abbreviations are used in this manuscript:

GCF	Gingival crevicular fluid
SOD	Superoxide dismutase
MDA	Malondialdehyde
8-OHdG	8-Hydroxy deoxyguanosine
GPx	Glutathione peroxidase
ELISA	Enzyme-linked immunosorbent assay
OHI	Oral hygiene index
GI	Gingival index
PBS	Phosphate saline buffer solution
EDTA	Ethylenediaminetetraacetic acid

## Appendix A

Descriptive statistics were expressed as mean ± standard error of the mean (SEM), and group differences were explored through a general linear model (GLM) framework. Given that the oxidative stress biomarkers were intercorrelated and measured in parallel biological matrices, a multivariate analysis of variance (MANOVA) was first applied to evaluate the overall effect of group (leukemia and control), oral hygiene, gingival inflammation, sex and age on the combined biomarker profile. The MANOVA design allowed for the simultaneous testing of multiple dependent variables while controlling for Type I error inflation and intercorrelation among outcomes. Among the multivariate test statistics available, Pillai’s Trace was selected as the main criterion because it is the most robust to violations of normality and unequal covariance matrices, especially in moderate sample sizes and unbalanced designs. When the overall multivariate test was significant or marginally significant, individual univariate GLMs were examined to identify specific effects on each biomarker. The Type II sums of squares option was used, as it provides unbiased estimates under unbalanced group sizes and partials out the effect of other factors before testing the variable of interest. For each model, the F-statistic, *p*-value, partial eta squared (η^2^*p*), and adjusted R^2^ were reported. Effect sizes were interpreted following Cohen’s conventions (small = 0.01, medium = 0.06, large = 0.14). Interactions between predictors (e.g., Gender × OHI, OHI × GI, Gender × GI) were included to explore potential moderating effects between oral health variables and demographic factors. Where interactions reached near-significance (*p* < 0.10), simple effects and estimated marginal means were examined to clarify directionality. Assumptions of normality and homoscedasticity were verified using Shapiro–Wilk and Levene’s tests, respectively. In case of variance heterogeneity, the robustness of Pillai’s Trace ensured reliable inference at the multivariate level. The results were visualized and summarized through estimated marginal means and 95% confidence intervals.

Since the primary objective of the study was comparative, analyses were performed on the original values of the biomarkers without logarithmic transformation. Although the distributions of some of the variables (e.g., 8-OHdG, MDA) show a slight asymmetry, GLM tests are considered robust to moderate deviations from normality, especially in the presence of balanced samples by group. In addition, tests for homogeneity of variances (Levene) and graphical inspection of residuals were used to verify the adequacy of the model. Additional analyses with logarithmic transformation were performed as a sensitivity check, the results maintaining the same trends, which is why the final reporting uses raw values for a straightforward clinical interpretation.

Given the exploratory nature of this study and the relatively small sample size, no formal correction for multiple testing (e.g., Holm–Bonferroni or FDR adjustment) was applied. Instead, we used a MANOVA/GLM framework to reduce the number of separate comparisons and to evaluate overall multivariate effects across biomarkers, followed by univariate tests for interpretative purposes. Therefore, the results should be interpreted with caution, acknowledging a potential inflation of type I error due to multiple comparisons. However, the consistency of group effects across related biomarkers (8-OHdG and SOD) and robustness in the sensitivity analysis support the reliability of the main findings.

**Table A1:** Results of the GLM test.

Tests of Between-Subjects Effects									
Source	Dependent Variable	Type II Sum of Squares	df	Mean Square	F	Sig.	Partial Eta Squared	Noncent. Parameter	Observed Power ^g^
Corrected Model	8-OHdG in GCF	117.536 ^a^	23	5.110	1.982	0.015	0.384	45.586	0.975
	8-OHdG in plasma	35,343.109 ^b^	23	1536.657	1.414	0.134	0.308	32.522	0.880
	SOD in GCF	1.923 ^c^	23	0.084	2.127	0.008	0.401	48.922	0.984
	SOD in plasma	2.385 ^d^	23	0.104	2.348	0.003	0.425	54.004	0.992
	MDA in GCF	99.690 ^e^	23	4.334	0.923	0.570	0.225	21.228	0.654
	MDA in plasma	138.048 ^f^	23	6.002	1.584	0.072	0.333	36.431	0.922
Intercept	8-OHdG in GCF	99.349	1	99.349	38.532	0.000	0.345	38.532	1.000
	8-OHdG in plasma	34,482.492	1	34,482.492	31.730	0.000	0.303	31.730	1.000
	SOD in GCF	451.852	1	451.852	11,494.414	0.000	0.994	11,494.414	1.000
	SOD in plasma	472.445	1	472.445	10,699.610	0.000	0.993	10,699.610	1.000
	MDA in GCF	18.864	1	18.864	4.017	0.049	0.052	4.017	0.507
	MDA in plasma	112.482	1	112.482	29.684	0.000	0.289	29.684	1.000
Gender	8-OHdG in GCF	0.443	1	0.443	0.172	0.680	0.002	0.172	0.069
	8-OHdG in plasma	115.685	1	115.685	0.106	0.745	0.001	0.106	0.062
	SOD in GCF	0.021	1	0.021	0.538	0.466	0.007	0.538	0.112
	SOD in plasma	0.004	1	0.004	0.079	0.779	0.001	0.079	0.059
	MDA in GCF	7.139	1	7.139	1.520	0.222	0.020	1.520	0.229
	MDA in plasma	1.123	1	1.123	0.296	0.588	0.004	0.296	0.084
Group	8-OHdG in GCF	24.171	1	24.171	9.375	0.003	0.114	9.375	0.856
	8-OHdG in plasma	4573.550	1	4573.550	4.209	0.044	0.055	4.209	0.526
	SOD in GCF	0.616	1	0.616	15.661	0.000	0.177	15.661	0.974
	SOD in plasma	0.925	1	0.925	20.938	0.000	0.223	20.938	0.995
	MDA in GCF	7.954	1	7.954	1.694	0.197	0.023	1.694	0.250
	MDA in plasma	12.619	1	12.619	3.330	0.072	0.044	3.330	0.437
Oral hygiene status	8-OHdG in GCF	9.280	3	3.093	1.200	0.316	0.047	3.599	0.309
	8-OHdG in plasma	2774.752	3	924.917	0.851	0.470	0.034	2.553	0.227
	SOD in GCF	0.101	3	0.034	0.854	0.469	0.034	2.563	0.227
	SOD in plasma	0.130	3	0.043	0.983	0.406	0.039	2.948	0.258
	MDA in GCF	18.955	3	6.318	1.345	0.266	0.052	4.036	0.344
	MDA in plasma	16.419	3	5.473	1.444	0.237	0.056	4.333	0.368
Gingival inflammation status	8-OHdG in GCF	2.069	2	1.035	0.401	0.671	0.011	0.803	0.113
	8-OHdG in plasma	297.244	2	148.622	0.137	0.872	0.004	0.274	0.070
	SOD in GCF	0.116	2	0.058	1.480	0.234	0.039	2.959	0.306
	SOD in plasma	0.040	2	0.020	0.451	0.639	0.012	0.901	0.121
	MDA in GCF	2.543	2	1.271	0.271	0.764	0.007	0.541	0.091
	MDA in plasma	3.604	2	1.802	0.475	0.623	0.013	0.951	0.125
Age	8-OHdG in GCF	0.576	1	0.576	0.223	0.638	0.003	0.223	0.075
	8-OHdG in plasma	934.280	1	934.280	0.860	0.357	0.012	0.860	0.150
	SOD in GCF	0.089	1	0.089	2.265	0.137	0.030	2.265	0.318
	SOD in plasma	0.006	1	0.006	0.132	0.717	0.002	0.132	0.065
	MDA in GCF	23.355	1	23.355	4.973	0.029	0.064	4.973	0.595
	MDA in plasma	0.373	1	0.373	0.098	0.755	0.001	0.098	0.061
Gender × Oral hygiene status	8-OHdG in GCF	14.816	3	4.939	1.915	0.135	0.073	5.746	0.476
	8-OHdG in plasma	3271.196	3	1090.399	1.003	0.396	0.040	3.010	0.262
	SOD in GCF	0.038	3	0.013	0.321	0.810	0.013	0.962	0.109
	SOD in plasma	0.345	3	0.115	2.603	0.058	0.097	7.810	0.616
	MDA in GCF	0.925	3	0.308	0.066	0.978	0.003	0.197	0.061
	MDA in plasma	6.588	3	2.196	0.580	0.630	0.023	1.739	0.164
Gender × Gingival inflammation status	8-OHdG in GCF	12.817	2	6.409	2.486	0.090	0.064	4.971	0.484
	8-OHdG in plasma	1285.530	2	642.765	0.591	0.556	0.016	1.183	0.145
	SOD in GCF	0.106	2	0.053	1.350	0.266	0.036	2.700	0.282
	SOD in plasma	0.041	2	0.021	0.466	0.629	0.013	0.932	0.123
	MDA in GCF	13.445	2	6.722	1.431	0.246	0.038	2.863	0.297
	MDA in plasma	5.361	2	2.681	0.707	0.496	0.019	1.415	0.165
Gender × Group	8-OHdG in GCF	0.055	1	0.055	0.021	0.884	0.000	0.021	0.052
	8-OHdG in plasma	9.919	1	9.919	0.009	0.924	0.000	0.009	0.051
	SOD in GCF	0.010	1	0.010	0.244	0.623	0.003	0.244	0.078
	SOD in plasma	0.037	1	0.037	0.839	0.363	0.011	0.839	0.148
	MDA in GCF	2.692	1	2.692	0.573	0.451	0.008	0.573	0.116
	MDA in plasma	0.390	1	0.390	0.103	0.749	0.001	0.103	0.062
Oral hygiene status × Gingival inflammation status	8-OHdG in GCF	12.700	6	2.117	0.821	0.557	0.063	4.926	0.305
	8-OHdG in plasma	13,516.861	6	2252.810	2.073	0.067	0.146	12.438	0.713
	SOD in GCF	0.124	6	0.021	0.526	0.787	0.041	3.153	0.200
	SOD in plasma	0.055	6	0.009	0.209	0.973	0.017	1.251	0.101
	MDA in GCF	12.407	6	2.068	0.440	0.850	0.035	2.642	0.171
	MDA in plasma	35.813	6	5.969	1.575	0.167	0.115	9.451	0.571
Group × Oral hygiene status	8-OHdG in GCF	1.618	2	0.809	0.314	0.732	0.009	0.628	0.098
	8-OHdG in plasma	864.815	2	432.407	0.398	0.673	0.011	0.796	0.112
	SOD in GCF	0.135	2	0.068	1.719	0.186	0.045	3.437	0.350
	SOD in plasma	0.056	2	0.028	0.632	0.534	0.017	1.265	0.152
	MDA in GCF	5.001	2	2.500	0.532	0.589	0.014	1.065	0.135
	MDA in plasma	7.776	2	3.888	1.026	0.364	0.027	2.052	0.223
Gender × Gingival inflammation status	8-OHdG in GCF	0.227	1	0.227	0.088	0.768	0.001	0.088	0.060
	8-OHdG in plasma	388.964	1	388.964	0.358	0.552	0.005	0.358	0.091
	SOD in GCF	0.094	1	0.094	2.398	0.126	0.032	2.398	0.333
	SOD in plasma	0.029	1	0.029	0.648	0.423	0.009	0.648	0.125
	MDA in GCF	2.852	1	2.852	0.607	0.438	0.008	0.607	0.120
	MDA in plasma	0.006	1	0.006	0.002	0.968	0.000	0.002	0.050
Error	8-OHdG in GCF	188.218	73	2.578					
	8-OHdG in plasma	79,331.324	73	1086.730					
	SOD in GCF	2.870	73	0.039					
	SOD in plasma	3.223	73	0.044					
	MDA in GCF	342.825	73	4.696					
	MDA in plasma	276.616	73	3.789					
Total	8-OHdG in GCF	674.121	97						
	8-OHdG in plasma	213,330.275	97						
	SOD in GCF	3722.163	97						
	SOD in plasma	3762.566	97						
	MDA in GCF	1021.669	97						
	MDA in plasma	968.590	97						
Corrected Total	8-OHdG in GCF	305.754	96						
	8-OHdG in plasma	114,674.434	96						
	SOD in GCF	4.793	96						
	SOD in plasma	5.608	96						
	MDA in GCF	442.515	96						
	MDA in plasma	414.664	96						

^a^ R Squared = 0.384 (Adjusted R Squared = 0.190); ^b^ R Squared = 0.308 (Adjusted R Squared = 0.090); ^c^ R Squared = 0.401 (Adjusted R Squared = 0.213); ^d^ R Squared = 0.425 (Adjusted R Squared = 0.244); ^e^ R Squared = 0.225 (Adjusted R Squared = −0.019); ^f^ R Squared = 0.333 (Adjusted R Squared = 0.123); ^g^ Computed using alpha = 0.05.
